# *In Vitro* Enhancement of Respiratory Syncytial Virus Infection by Maternal Antibodies Does Not Explain Disease Severity in Infants

**DOI:** 10.1128/JVI.00851-17

**Published:** 2017-10-13

**Authors:** Elisabeth A. van Erp, Puck B. van Kasteren, Teun Guichelaar, Inge M. L. Ahout, Cornelis A. M. de Haan, Willem Luytjes, Gerben Ferwerda, Oliver Wicht

**Affiliations:** a Centre for Infectious Disease Control, National Institute for Public Health and the Environment (RIVM), Bilthoven, The Netherlands; b Laboratory of Pediatric Infectious Diseases, Department of Pediatrics, Radboud Institute for Molecular Life Sciences, Radboud University Medical Center, Nijmegen, The Netherlands; c Virology Division, Department of Infectious Diseases and Immunology, Faculty of Veterinary Medicine, Utrecht University, Utrecht, The Netherlands; Hudson Institute of Medical Research

**Keywords:** antibody-dependent enhancement, maternal antibodies, antibody function, cotton rat, neonatal immunology, neutralizing antibodies, pediatric infectious disease, respiratory syncytial virus, virology

## Abstract

Respiratory syncytial virus (RSV) is the leading cause of severe respiratory illness in infants. At this young age, infants typically depend on maternally transferred antibodies (matAbs) and their innate immune system for protection against infections. RSV-specific matAbs are thought to protect from severe illness, yet severe RSV disease occurs mainly below 6 months of age, when neutralizing matAb levels are present. To investigate this discrepancy, we asked if disease severity is related to antibody properties other than neutralization. Some antibody effector functions are mediated via their Fc binding region. However, it has been shown that this binding may lead to antibody-dependent enhancement (ADE) of infection or reduction of neutralization, both possibly leading to more disease. In this study, we first showed that high levels of ADE of RSV infection occur in monocytic THP-1 cells in the presence of RSV antibodies and that neutralization by these antibodies was reduced in Vero cells when they were transduced with Fc gamma receptors. We then demonstrated that antibodies from cotton rats with formalin-inactivated (FI)-RSV-induced pulmonary pathology were capable of causing ADE. Human matAbs also caused ADE and were less neutralizing *in vitro* in cells that carry Fc receptors. However, these effects were unrelated to disease severity because they were seen both in uninfected controls and in infants hospitalized with different levels of RSV disease severity. We conclude that ADE and reduction of neutralization are unlikely to be involved in RSV disease in infants with neutralizing matAbs.

**IMPORTANCE** It is unclear why severity of RSV disease peaks at the age when infants have neutralizing levels of maternal antibodies. Additionally, the exact reason for FI-RSV-induced enhanced disease, as seen in the 1960s vaccine trials, is still unclear. We hypothesized that antibodies present under either of these conditions could contribute to disease severity. Antibodies can have effects that may lead to more disease instead of protection. We investigated two of those effects: antibody-dependent enhancement of infection (ADE) and neutralization reduction. We show that ADE occurs *in vitro* with antibodies from FI-RSV-immunized RSV-infected cotton rats. Moreover, passively acquired maternal antibodies from infants had the capacity to induce ADE and reduction of neutralization. However, no clear association with disease severity was seen, ruling out that these properties explain disease in the presence of maternal antibodies. Our data contribute to a better understanding of the impact of antibodies on RSV disease in infants.

## INTRODUCTION

Human respiratory syncytial virus (RSV) is the leading cause of lower respiratory tract disease in young children (1). Hospitalization for severe RSV-mediated disease is most frequent between 6 weeks and 6 months of life (2, 3). It seems widely accepted that RSV-neutralizing, transplacentally transferred maternal antibodies (matAbs) can lower the risk for RSV infections (1, 4, 5); however, many studies fail to reproduce these results (6–8). There is no consensus on what level of antibodies is sufficient for protection, but an average concentration of maternal antibodies seems insufficient.

Maternal antibody levels are high during the first 6 months after birth. Strikingly, severe RSV disease most frequently occurs in this period of life (2, 3), indicating RSV can infect infants even though matAbs are present. For these children who become infected with RSV, the role of matAbs in RSV disease is unclear. Associations between the severity of symptoms and classical serological parameters such as RSV neutralization titer or RSV-specific antibody levels have not been observed so far (4, 6). However, antibodies have additional effector functions, generally mediated via their Fc binding region, that contribute to immune defense. In this study, we investigate whether consequences of antibody-Fc receptor interactions could be related to the severity of symptoms during RSV infection.

Antibody-dependent enhancement (ADE) of RSV infection has been demonstrated *in vitro* (9) in monocytic cell lines. ADE means that RSV-specific antibodies in human serum, as well as monoclonal antibodies, increase the number of RSV-infected cells when those cells carry Fc gamma receptors (FCGR) (10, 11). FCGR-carrying leukocytes, which are present in the lungs or recruited during infection, might be more readily infected and activated by RSV-antibody complexes than RSV alone. In addition to causing ADE, Fc receptors lower the RSV neutralization capacity of most monoclonal antibodies (12), allowing RSV to infect even in the presence of otherwise neutralizing antibody titers. Whether FCGR binding influences RSV (immuno-)pathology and severity of RSV disease has not been studied so far, but the ongoing pursuit of RSV vaccines demands more knowledge about the role of antibodies in RSV disease. Therefore, we investigated whether reduced neutralization and enhanced RSV infection of FCGR-bearing cells by maternal antibodies that should provide passively acquired immunity relates to clinical disease severity in infants. Moreover, antibodies generated as part of active immunity induced by formalin-inactivated (FI)-RSV vaccination have been shown to contribute to enhanced pathology (13). Therefore, we also investigated whether reduced neutralization and enhanced RSV infection of FCGR-bearing cells relates to pathology in the FI-RSV cotton rat model.

We assessed two FCGR-mediated effects of RSV-specific antibodies: antibody-dependent enhancement of infection in monocytic THP-1 cells and reduction of neutralization capacity in cells transduced with Fc gamma receptors. Both were investigated for passively transferred matAbs by titration series of plasma from human cord blood and for actively acquired antibodies from immunized cotton rats, a small-animal model that is highly susceptible to RSV and shows vaccine-enhanced pulmonary pathology (14, 15). To assess possible associations with disease severity, plasma samples from infants with acute primary RSV infection were tested, as were sera from cotton rats showing FI-RSV-induced enhancement of pathology.

## RESULTS

### ADE of RSV infection and ΔPRNT50.

Infants that encounter RSV in the first months of life have maternal antibodies. Despite the presence of maternal antibodies, a subset of infants that encounter RSV become severely ill. The antibodies of severely diseased infants are not neutralizing enough to prevent infection, but they can induce multiple other effector functions through FCGR interactions. In this paper, we investigate the impact of these Fc receptor-mediated functions of maternal antibodies on the severity of disease symptoms during RSV infection. We assessed two antibody-Fc receptor-mediated effects *in vitro*: antibody-dependent enhancement (ADE) and reduction of RSV neutralization (change in 50% plaque reduction neutralization titers, or ΔPRNT50).

**FIG 1:**
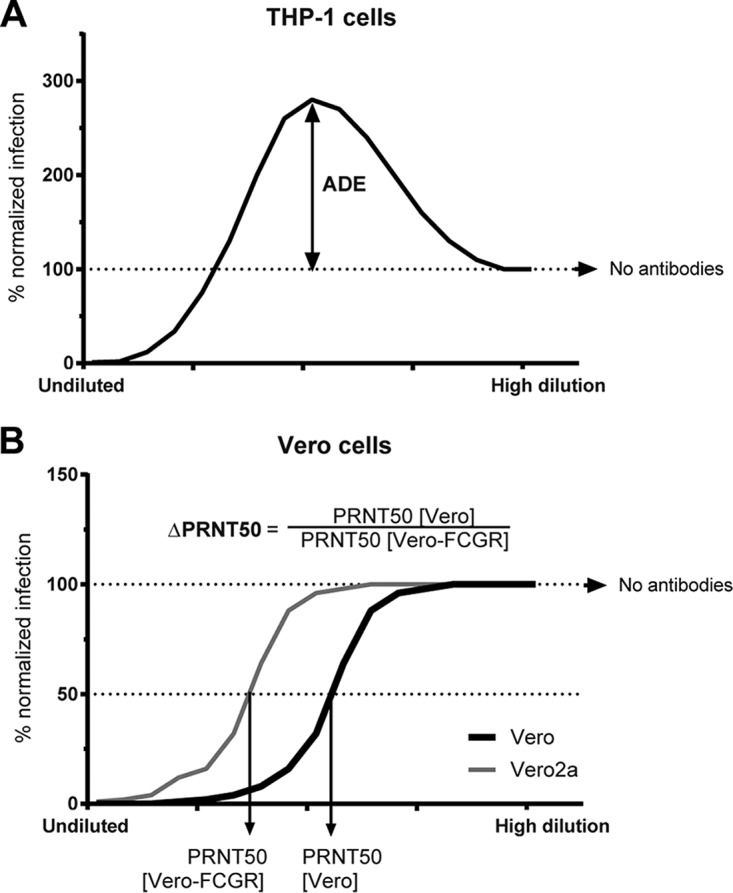
Antibody-dependent enhancement (ADE) and reduction of neutralization (ΔPRNT50) of RSV infection. Schematic figure depicting the calculation of ADE in THP-1 cells (A) and ΔPRNT50 between WT and FCGR-transduced Vero cells (B).

ADE of RSV infection in the presence of human serum or RSV-specific monoclonal antibodies has been demonstrated previously in monocytic cell lines, which carry Fc receptors (10, 11). ADE of RSV infection can be induced by subneutralizing concentrations of antibodies. At low concentrations, antibodies are not able to neutralize infection but bind RSV and mediate binding to Fc receptors. This attachment and subsequent internalization facilitates higher infection rates than those in the absence of antibodies and is schematically illustrated for monocytic THP-1 cells in Fig. 1A.

The second effect we investigated is the ability of the FCGR to reduce the neutralization capacity (PRNT50) of antibodies. Antibodies have higher neutralization potential on cells lacking FCGR than the same cell type expressing FCGR, as is shown for wild-type Vero cells compared to Vero cells expressing FCGR (12). We called this change in neutralization mediated by FCGR ΔPRNT50 throughout the manuscript (Fig. 1B). ΔPRNT50 represents the factor of reduction of neutralization and is calculated by dividing the PRNT50 in Vero cells by the PRNT50 in Vero cells expressing FCGR2a (Vero2a).

### ADE in primary cells and monocytic cell line THP-1.

**FIG 2:**
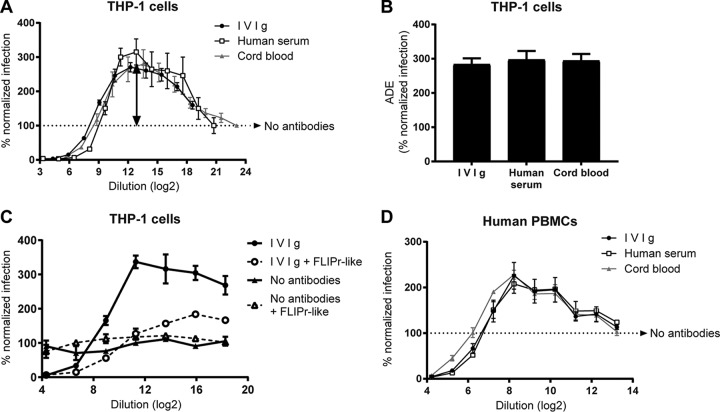
Antibody-dependent enhancement in monocytic cell line THP-1 and primary cells. (A) RSV neutralization assay using FCGR-carrying THP-1 cells in the presence of serially diluted IVIg, human serum, or cord blood. (B) ADE is reported for the dilution at which infection is maximal (depicted by the arrow in panel A). Fc-binding sites of FCGRs were blocked by adding 3 μg/ml staphylococcal FLIPr-like protein to THP-1 cells during RSV neutralization assay. (D) RSV neutralization assay using cells that were isolated from human blood (human PBMCs). One representative graph of three PBMC donors is depicted. For other graphs, the means and standard deviations (SD) from ≥3 individual experiments are shown.

ADE of RSV infection in the presence of human serum has been demonstrated previously (10). To investigate the effect of serum antibodies only and exclude the effect of other serum components, we used purified human immunoglobulin (IVIg). Serial dilutions of IVIg neutralized RSV (0% normalized infection) at high antibody concentrations and then facilitated up to 300% infection compared to the condition without antibodies (100% normalized infection) in monocytic, FCGR-carrying THP-1 cells (Fig. 2A, arrow points to maximum infection enhancement). Commercially available pooled human AB serum and a pool of cord blood plasma, both containing nonimmunoglobulin components, showed similar titration curves. Maximum ADE was similar for the three antibody solutions (Fig. 2B). To confirm that ADE is mediated by antibody-FCGR interactions in THP-1 cells, FLIPr-like protein was supplemented, which blocks FCGR-mediated Fc binding. FLIPr-like protein reduced ADE from 340% to 125% infection for IVIg (Fig. 2C). To assess whether ADE can occur in primary cells, we performed an ADE assay using human PBMCs. Also in primary immune cells, RSV infection was enhanced up to 230% (Fig. 2D).

### ΔPRNT50 on FCGR2a-expressing Vero cells as measure for FCGR-mediated reduction of neutralization.

**FIG 3:**
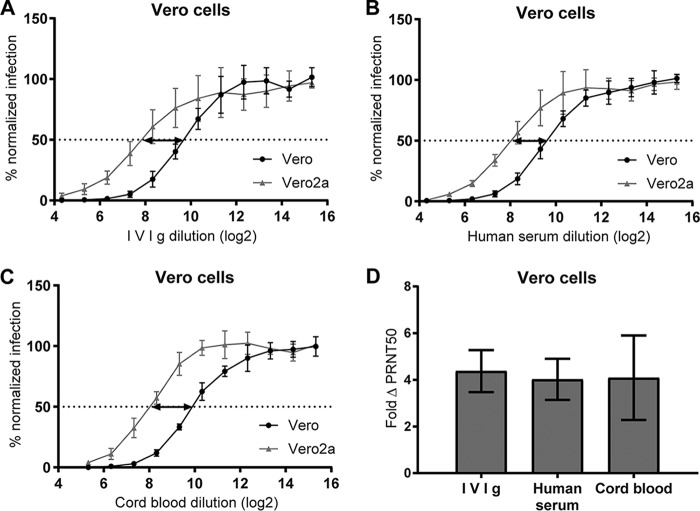
Reduction of neutralization of RSV in FCGR2a-transduced Vero cells. (A to C) RSV neutralization assays using parental and FCGR2a-transduced Vero cells (Vero2a) in the presence of serially diluted IVIg, human serum, or cord blood. (D) The ΔPRNT50 is a measure for the reduction of neutralization titer in Vero2a cells calculated by dividing PRNT50[Vero] by PRNT50[Vero2a] (depicted by the arrows in panels A to C). Means and SD from ≥3 individual experiments are shown.

Eliciting RSV-neutralizing antibodies is the primary goal of RSV vaccination efforts. Intriguingly, the presence of FCGRs in target cells mostly reduces the RSV neutralization capacity of monoclonal antibodies (12). Preventing Fc receptor interactions restored RSV neutralization by IVIg in THP-1 cells (Fig. 2C). We previously demonstrated that the impact of FCGRs on RSV neutralization can be measured in Vero cells that stably express FCGR2a (Vero2a) (12). In this study, titration series of IVIg, pooled human AB serum, and pooled cord blood plasma revealed a 4-fold-reduced neutralization on Vero2a compared to parental Vero cells (Fig. 3A to D). Although reduction in neutralization can be measured, FCGR-expressing Vero cells show no ADE, which is in line with observations for dengue virus (DenV) (16).

### Interindividual variation in ADE and ΔPRNT50.

**FIG 4:**
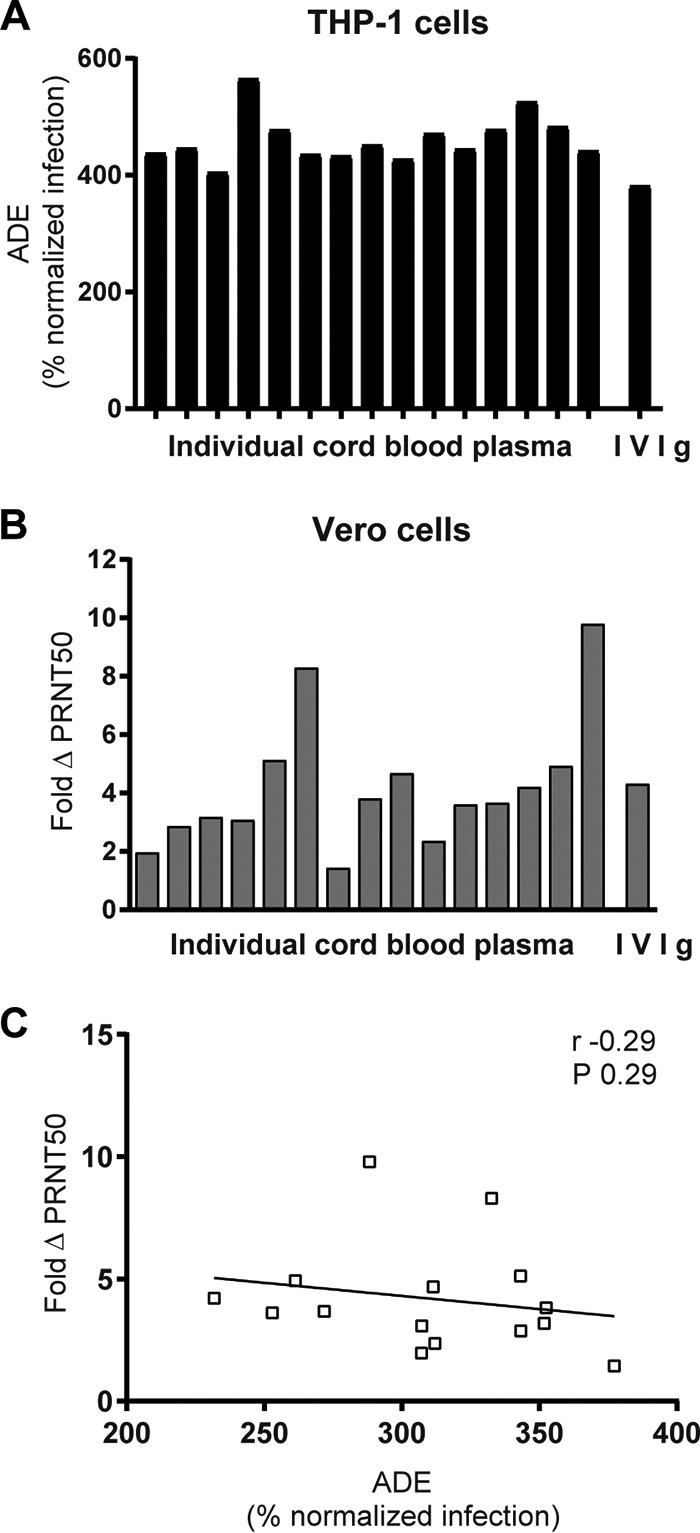
ADE and ΔPRNT50 by maternal antibodies. ADE (A) and ΔPRNT50 (B) were determined for 15 individual cord blood samples. (C) Statistical dependence between ADE and ΔPRNT50 was tested by Spearman's correlation.

To further analyze ADE or ΔPRNT50 and any link to disease symptoms, we first assessed whether it would be feasible to distinguish between individuals with different capacities of ADE and ΔPRNT50. We tested the two Fc-mediated effects in cord blood from 15 individual donors. Most samples showed about 400% ADE compared to infection in the absence of antibodies (Fig. 4A). The ΔPRNT50 showed a larger individual variation with up to 10-fold reduction of RSV neutralization in Vero2a cells (Fig. 4B). Interestingly, the ΔPRNT50 was unrelated to ADE (Fig. 4C), indicating that they are independent effects.

### ADE and ΔPRNT50 do not correlate with RSV neutralization or anti-RSV IgG levels.

**FIG 5:**
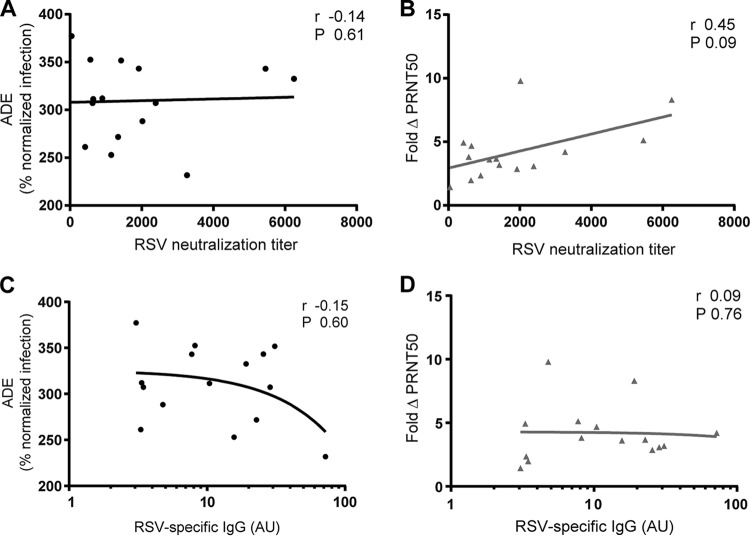
ADE and ΔPRNT50 are independent of classical serological parameters. ADE and ΔPRNT50 of 15 individual cord blood samples were compared to the RSV neutralization titer in Vero cells (A and B) or the relative amount of RSV-specific IgG (C and D). Associations were tested with Spearman's correlation.

To investigate a possible contribution of classical antibody characteristics to ADE or ΔPRNT50, both phenotypes were compared to two classical serological parameters: RSV neutralization in Vero cells (Fig. 5A and B) and amount of RSV-specific IgG (Fig. 5C and D). Slightly more reduction of neutralization was detected when RSV neutralization titers were highest (Fig. 5B), but this was not significant. No associations between any of the other parameters were detected.

### ADE in primary cells from cotton rat lungs.

**FIG 6:**
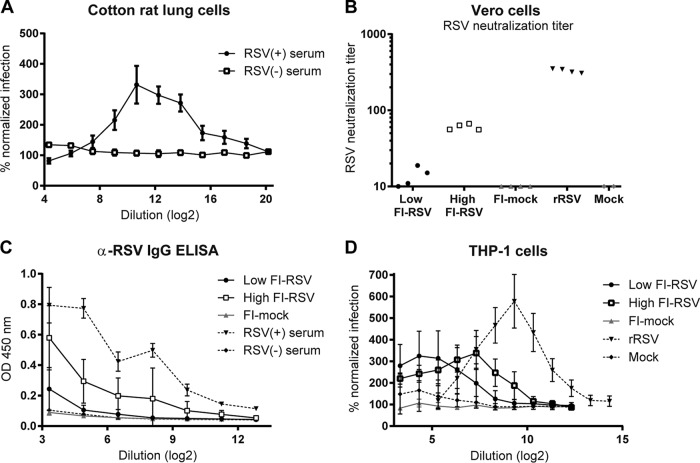
ADE by RSV-specific cotton rat antibodies. (A) RSV neutralization assay using cells that were isolated from cotton rat lungs. Plasma pools were from naive (−) or wild-type RSV-infected (+) cotton rats. (B to D) Four animals per group were immunized with live RSV (rRSV) or medium (mock) or with a low- or high-dose FI-RSV or formalin-treated medium (FI-mock) and subsequently challenged with RSV. (B and C) RSV neutralization titer (B) and RSV-specific IgG (C) of cotton rat plasma on day 4 after challenge. OD 450 nm, optical density at 450 nm. (D) RSV neutralization assay using THP-1 cells to determine the maximum ADE of cotton rat plasma.

ADE of RSV infection has not been demonstrated *in vivo*, but the inoculation of mice with RSV in complex with antibodies has been shown to reshape the immune response compared to RSV alone (17). Natural RSV infections target pulmonary tissue; therefore, we tested cells derived from lungs of naive cotton rats. To determine the type of cells in these lung cell isolates, we stained the cells for the epithelial marker pancytokeratin (18) and T cell marker CD3ε (19). Only 3% of cells were of epithelial origin, whereas 35% of cells were CD3ε positive (data not shown). This indicated that a large proportion of isolated cells were immune cells. Pools of plasma from RSV-challenged animals enhanced RSV infection in these cells by 330% (Fig. 6A). No ADE was induced when serum from naive cotton rats was used, indicating the requirement of RSV-specific antibodies for ADE.

### Characterization of FI-RSV-induced antibodies in immunized cotton rats.

As cotton rat antibodies could enhance RSV infection in primary cells, we wanted to understand their effect on RSV disease. It has been shown that antibodies generated as part of active immunity induced in infants by FI-RSV vaccination contribute to enhanced pathology (13). The cotton rat provides a model for FI-RSV-induced RSV pulmonary pathology (15). Therefore, we investigated whether reduced neutralization and enhanced RSV infection of FCGR-bearing cells relates to pathology in the FI-RSV cotton rat model.

Animals were immunized with FI-RSV for nonprotective antibodies or recombinant live-attenuated RSV (rRSV) for protective immunity. First, RSV-specific antibodies in cotton rats were characterized. RSV neutralization in Vero cells demonstrated low, dose-dependent titers of neutralizing antibodies in all FI-RSV-immunized animals, whereas neutralization capacity of plasma from animals immunized with rRSV was high (Fig. 6B). Correspondingly, RSV-specific antibody levels were low after FI-RSV and high after rRSV immunization (Fig. 6C).

The ability of cotton rat antibodies to cause ADE in the human THP-1 cell model was assessed next. Plasma of all immunized animals caused ADE in THP-1 cells (Fig. 6D). Infection was increased by 580% for plasma from rRSV-immunized animals and 380% after FI-RSV immunization. The dilution at which maximum ADE was measured was low for low-dose FI-RSV-, intermediate for high-dose FI-RSV-, and highest for rRSV-immunized animals. This corresponds to RSV neutralization titers and RSV-specific antibody levels. ΔPRNT50 could not be determined for the cotton rat sera due to a lack of neutralizing capacity, making it impossible to calculate a PRNT50.

### Serum of cotton rats with lung pathology causes ADE in the absence of neutralization.

**FIG 7:**
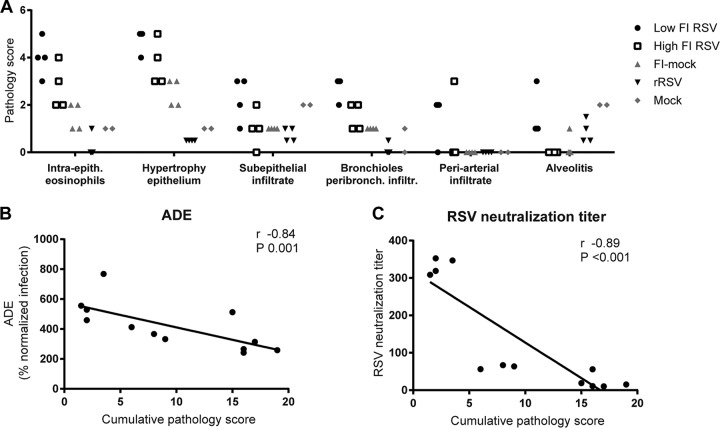
Lung pathology in cotton rats that show ADE *in vitro* in the absence of neutralization. Four animals per group were immunized with live RSV (rRSV) or medium only (mock) or with low- or high-dose FI-RSV or formalin-treated medium (FI-mock) and subsequently challenged with RSV. Intra-epith., intraepithelial; peribronch. infiltr., peribronchial infiltrate. (A) Histopathologic lung pathology scores. (B and C) Cumulative pathology scores, calculated by the sum of individual pathology scores, were compared to ADE and PRNT50 by Spearman's correlation.

To assess the relationship between ADE of RSV infection and severity of pulmonary pathology in cotton rats, pathology after RSV challenge of FI-RSV- or rRSV-immunized cotton rats was studied. Affirming previous studies, immunization with rRSV protected the animals and resulted in little pathology after challenge (Fig. 7A). Both FI-RSV-immunized groups showed elevated lung pathology compared to the group treated with rRSV or the mock vaccine. Moreover, the level of subepithelial, periarterial, and bronchiolar infiltrate was higher in FI-RSV-immunized cotton rats than in the control groups in our study. This is in accordance with the increased lymphocyte infiltration in FI-RSV-vaccinated children and animals in previous studies (20, 21).

The ADE and RSV neutralization titers of cotton rat sera next were compared to the sum of pathology scores (cumulative pathology score). Sera from mock-immunized animals were excluded. The capacity to induce ADE was present in all sera that contained RSV-specific antibodies independent of the pathology score. More ADE appears to be linked to lower levels of pathology, although the difference in ADE over the range of pathology was small (Fig. 7B). A lower pathology score might concur with efficient RSV neutralization and high anti-RSV antibody levels (Fig. 6B and C). The latter was confirmed, as pathological consequences of RSV challenge were high when RSV neutralization titers were low (Fig. 7C). Thus, antibodies from cotton rats with FI-RSV-induced pulmonary pathology were weak neutralizers and capable of causing ADE *in vitro*.

### ADE and ΔPRNT50 do not correlate with RSV disease outcome in hospitalized infants.

As ADE measured *in vitro* might relate to severe disease *in vivo* in the absence of neutralization, a possible association between severity of symptoms in infants and ADE or ΔPRNT50 was investigated. Plasma from hospitalized patients with an acute primary RSV infection was analyzed. As this is not a prospective study, the ideal control group of RSV-infected children with mild symptoms was not available, because blood of these children is not routinely sampled. A common practice in RSV research is to use age-matched uninfected infants as controls. One assumes that this group is not protected and if healthy infants become RSV infected, a vast majority (98 to 99%) would develop only mild symptoms (22). Analysis of the plasma samples showed that RSV neutralization and epitope specificity did not correlate with severity of symptoms (6). The lack of this correlation indicates there is no bias for antibody concentration or neutralization in each disease group. Therefore, this is an ideal sample set to assess ADE and ΔPRNT50 on FCGR-expressing cells.

**FIG 8:**
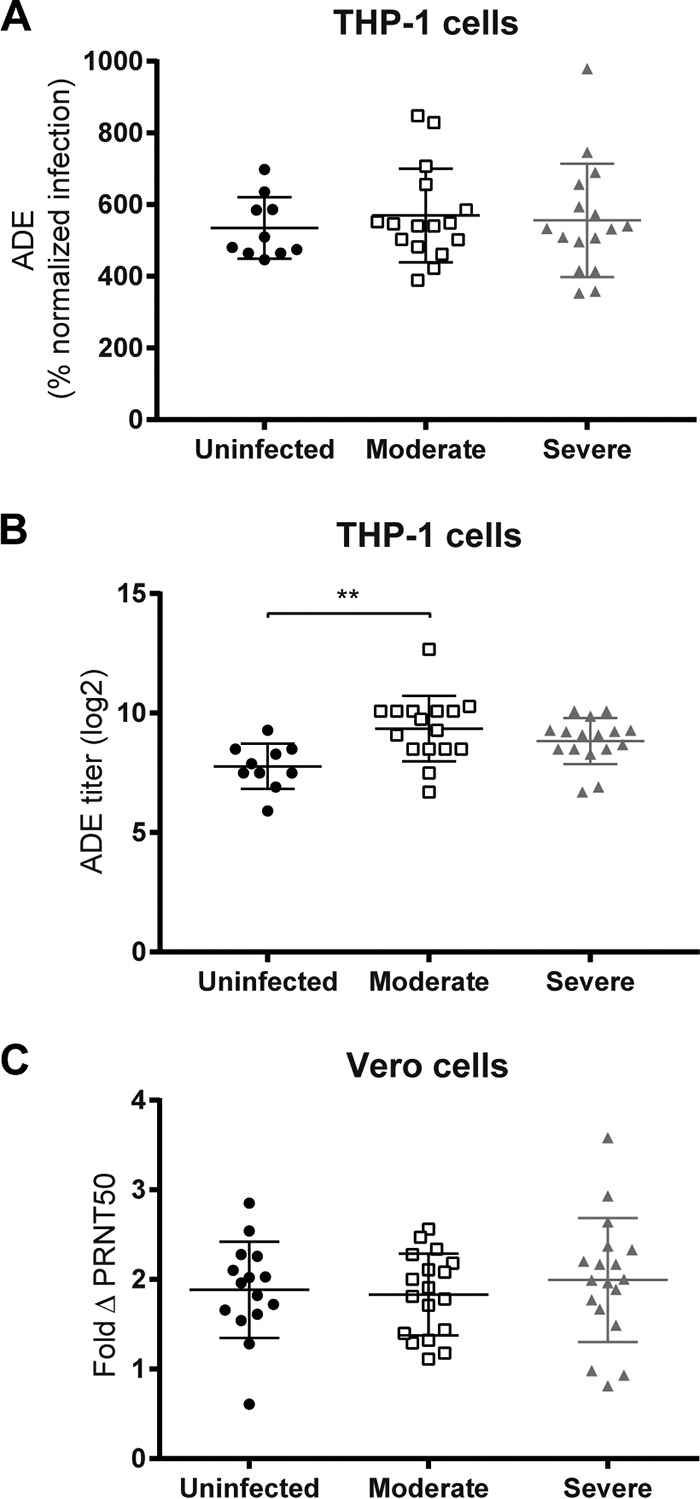
ADE and ΔPRNT50 by plasma from patients with different severities of RSV-mediated disease symptoms. RSV neutralization assays using plasma samples from patients with moderate or severe RSV-mediated disease and healthy control patients (uninfected). Maximum ADE (A) and plasma titer at which ADE was maximal (B) were compared to disease severity. (C) ΔPRNT50 was compared to disease severity. Statistical analyses employed one-way analysis of variance for comparison between the three disease groups (**, *P* < 0.01).

First, maximum ADE of RSV infection by plasma from uninfected, moderate, and severe disease groups was examined on THP-1 cells. ADE ranged from 400 to 1,000% of infection, showing a greater variation than that in cord blood samples. However, disease severity and average ADE were not related (Fig. 8A). The plasma titer at which ADE was maximal next was calculated for all samples. The infants with moderate RSV disease had a significantly higher ADE titer than the uninfected controls, but no correlation with severe disease could be found (Fig. 8B). On Vero2a cells, the ΔPRNT50 ranged from 1- to 4-fold-reduced neutralization (Fig. 8C), but the average ΔPRNT50 was similar in all groups. We also checked correlation between ADE, ΔPRNT50, and age, but no relation was found (data not shown).

## DISCUSSION

Serum concentration and neutralization capacity are of primary concern when assessing the antiviral activity of antibodies. However, antibodies have many other functions, mediated by the Fc region, that play a role in the immune response. On FCGR-bearing cells, the antibody-FCGR interaction may lead to antibody-dependent enhancement (ADE) of infection or to reduction of neutralization (ΔPRNT50). We performed this study because we were intrigued by the fact that most severe RSV cases occur in RSV patients under the age of 6 months, when relatively high levels of RSV-neutralizing matAbs can be found. Moreover, infants that experienced vaccine-enhanced disease after the 1960s FI-RSV trials had an RSV-specific antibody response, albeit nonprotective. Therefore, both of these maternal and FI-RSV-induced antibodies could have a detrimental role in RSV-mediated disease when not neutralizing enough to protect infants from infection.

Generally, Fc-mediated effector functions of RSV-specific antibodies are not considered when studying acute RSV disease, even though some studies demonstrated ADE of RSV infection *in vitro*, including an increase of ADE after RSV infection (9–11, 23). RSV-specific matAbs may cause ADE and reshape the immune response against RSV (17, 23, 24). Interestingly, matAbs against dengue virus (DenV) have been suggested to worsen DenV disease in infants (25, 26).

We investigated whether FCGR interactions of RSV-specific serum antibodies relate to the severity of RSV disease symptoms in different settings. ADE of RSV infection and ΔPRNT50 were studied in FCGR-bearing cells using plasma from naive children with severe RSV disease, cord blood plasma, and plasma from FI-RSV-immunized cotton rats showing pathology. There are clear differences between severe RSV disease in naive infants, which fully depend on maternal antibodies for RSV-specific immunity, and FI-RSV vaccine-induced pathology, which is mediated by pathogenic vaccine-induced antibodies in the presence of an unbalanced cellular response (27). For example, FI-RSV vaccine-enhanced pulmonary pathology is associated with inadequate cellular immune responses (28–30). Another well-established difference is the low RSV neutralization capacity of serum antibodies induced by FI-RSV vaccination (13, 31, 32). The use of sera from two different types of RSV disease allowed us to assess the ADE and neutralization-reducing characteristics for both passively acquired matAbs and actively acquired antibodies in a vaccination setting.

ADE was seen for plasma of both healthy and severely ill infants in different degrees, but we did not find a correlation between ADE and RSV disease. There are several considerations to be made. First, it is well described that ADE is related to more disease severity in DenV (33–35). However, there are fundamental differences between DenV and RSV infection. Only a single serotype exists for RSV, whereas heterotypic, nonneutralizing antibodies exacerbate DenV disease. In addition, the primary target cells for RSV are lung epithelial cells, in contrast to immune cells for DenV. Interestingly, there is a growing body of evidence that RSV also infects immune cells. Recently, infection of several subsets of immune cells has been found in infants with severe RSV disease (36, 37). Infection of these FCGR-carrying immune cells may well be aided by ADE, which could affect the immune response and subsequent pathology, as in DENV infections.

Second, our study demonstrates that ADE of RSV *in vitro* occurs only at nonneutralizing concentrations of antibodies. Even minute amounts of antibodies that fail to neutralize can mediate ADE. Such conditions may have existed in some of the infants in our study. Bronchial antibody concentrations can be about 100 times lower than those in circulation, where we measured them (38–40), and RSV-specific matAbs can decline rapidly, with a half-life of approximately 1 month (3). Such an age-dependent decline of matAbs has been described in DenV infections and has been suggested to increase the risk for severe disease (41, 42). Interestingly, in contrast to most RSV-specific monoclonal antibodies, neutralization by palivizumab improves in cells expressing FCGR (12). This unique feature of the therapeutic monoclonal antibody might explain its clinical efficacy.

Third, our findings on maternal antibodies from children with various levels of RSV disease were different from findings on antibodies from cotton rats that show vaccine-enhanced pulmonary pathology mediated by FI-RSV-induced immune responses. In children, neither ADE nor reduced neutralization correlated with disease severity, but in FI-RSV-immunized cotton rats high ADE capacity *in vitro* coincides with pathology in the absence, but not in the presence, of neutralization, as seen for rRSV-immunized animals. This suggests that ADE cannot induce severe pathology in the presence of neutralization but should be considered under nonneutralizing conditions.

Although various reports do emphasize the importance of antibodies in vaccine-enhanced RSV disease (13, 31, 32, 43), it is generally accepted that FI-RSV vaccine-enhanced pathology results from an unbalanced immune response and a combination of aberrant humoral and cellular responses. In animal models, CD4^+^ T cells seem to be especially important mediators of pathology (28, 30). Interestingly, such a requirement for the FCGR-carrying cellular arm of the immune system might explain why RSV-specific antibodies have a low probability of influencing disease through ADE in the absence of a cellular response, as is the case with maternally derived antibodies. It would also be in accordance with data from a previous study in which it was shown that passive transfer of serum from FI-RSV-immunized cotton rats to naive animals was not sufficient to induce vaccine-enhanced disease (44). This does not exclude that such antibodies in their original setting (i.e., acquired through immunization) and in combination with immune cells could play a role in disease, but only when they are nonneutralizing, as is the case in FI-RSV-immunized animals.

Our data show that severe pulmonary pathology in the cotton rat FI-RSV vaccination model occurs in the presence of antibodies that are nonneutralizing and cause ADE *in vitro*. This relation may also exist in humans: since the initial demonstration that the formalin-inactivated vaccine enhanced disease severity in children, ADE has been considered an explanation (45). FI-RSV-immunized infants developed nonneutralizing antibody titers, a prerequisite for ADE, possibly because formalin treatment of RSV modified the viral surface epitopes (11, 46). Correspondingly, vaccine-enhanced pathology was partially attributed to increased levels of nonneutralizing antibodies and immune complex deposition in previous reports (13, 31, 32, 43). This could lead to augmented RSV infection of immune cells that carry FCGR and/or reshaping of the immune response by FCGR signaling.

Recently, it has been shown that binding of immune complexes to FCGR is not enough to induce ADE for Ebola virus (47). Downstream signaling pathways were required to observe enhancement of infection. This may explain why we were unable to detect ADE on the FCGR-transduced Vero cells. Most likely, the FCGRs expressed on Vero cells are nonfunctional in signaling; they only mediate the binding of immune complexes and cannot internalize the RSV-antibody immune complexes.

We show that there is ADE capacity *in vitro* when RSV-mediated pathology and RSV-specific antibodies are present. However, additional research is needed to elucidate the involvement of ADE in infection of immune cells *in vivo* during natural RSV infection or after vaccination. We did not see a correlation between ADE *in vitro* and disease severity for the infants in our cohort with the assays described in this paper. However, multiple additional antibody functions that may have immunomodulatory effects have not been tested yet (reviewed in reference 48). In addition, using THP-1 cells as a model system only demonstrates the effect on monocytes, whereas other immune cells express different combinations of FCGRs and could react differently. It could well be that ADE is part of a set of conditions, including the status of the infant's immune system or airway size, that determine the outcome of RSV infection.

In summary, antibodies can favor ADE and have reduced neutralization capacity in Fc receptor-carrying cells. Antibodies from FI-RSV-immunized cotton rats showed no neutralization and were able to induce ADE *in vitro*. In infants, ADE and reduced neutralization by matAbs *in vitro* did not relate to severity of RSV disease symptoms, although all tested human plasma had the capacity to cause ADE. There may be different effects from passively transferred matAbs compared to antibodies induced by direct vaccination, particularly when they are nonneutralizing, as we show for those induced by formalin-inactivated vaccines. This could lead to different outcomes of ADE.

This report shows that ADE should not be ignored as a possible player in RSV infection. Most importantly, the induction of nonneutralizing antibodies or subneutralizing antibody titers should be avoided. It remains a challenge to find out whether immune cells are infected through ADE of RSV infection *in vivo*. It should also be noted that RSV infection of immune cells may have multiple effects, ranging from (hyper)activation to shutting down cellular effector functions. Any of these effects could lead to the known contribution of immune cells to immunopathology in severe RSV-mediated disease. Determining whether ADE of RSV infection is possible in these cells *in vivo* and how these cells react may provide clues to why severe RSV disease occurs in certain cases and how we can prevent the burden of disease in these young infants.

## MATERIALS AND METHODS

### Study design.

Plasma samples from healthy controls and hospitalized infants with RSV infections used in this study have been described before (6). Hospitalized children below 1 year of age with PCR-confirmed RSV infections were included during 2011 to 2013. In the previous study, only samples from children below 3 months of age were included (6). In this study, we included all 51 children, of which 20% were >3 months of age. Blood samples were taken within 24 h after admission. Patients with congenital heart or lung disease, immunodeficiency, or glucocorticoid use and infants born at a gestational age below 35 weeks were excluded. For analysis, patients were classified with severe symptoms when they required mechanical ventilation and admission to the intensive care unit. Infants with moderate disease were only monitored or received oxygen therapy. Infants below 1 year of age requiring surgery for an inguinal hernia repair were included as healthy controls. Nasopharyngeal aspirates from all uninfected individuals were RSV negative. Gender, gestational age, presence of breastfeeding, and presence of parental smoking were comparable between all groups. The study protocols were approved by the Regional Committee on Research Involving Human Subjects Arnhem-Nijmegen (serving as the IRB) and were conducted in accordance with the principles of the Declaration of Helsinki. Written informed consent was obtained from the parents of all infants. A potential limitation of our study is the difficulty of separating the presence of matAbs from endogenous RSV-induced antibodies of the infant. However, with a median onset of disease of 3 days and the short time between symptoms and hospitalization, production of endogenous RSV-IgG is unlikely to have reached significant levels at the time of sampling. About 80% of patients were below 3 months of age. As Dutch infants in general suffer their first respiratory infection after 3 to 7 months of age, infants in our study most likely experienced a primary RSV infection (49). Therefore, it is plausible that we detected mainly matAbs (50).

### Cells.

Monkey kidney epithelial Vero-CCL81 cells were propagated in Dulbecco's modified Eagle's medium (DMEM) supplemented with 5% fetal calf serum (FCS) and 1% penicillin–streptomycin–GlutaMAX (PSG). The Vero cell-derived cell line expressing FCGR2a (Vero2a) was described earlier (12), and FCGR2a expression was confirmed by flow cytometry (data not shown). Monocytic THP-1 cells were cultured in RPMI supplemented with 10% FCS and PSG (51). Peripheral blood mononuclear cells (PBMC) were obtained from healthy volunteers. Blood was collected in heparin tubes, and the PBMC fraction was isolated by density gradient centrifugation using Lymphoprep (Nycomed).

### Virus and vaccine preparation.

Live-attenuated recombinant RSV-X (rRSV) (52), recombinant RSV-X containing a green fluorescent protein (GFP) gene (designated RSV [52]), and RSV-A2 were propagated in Vero cells as described before (52) (Dutch GMO license IG-99-210). Virus stocks were purified between layers of 10% and 50% sucrose by ultracentrifugation. FI-RSV and formalin-inactivated cell culture supernatant were prepared from RSV-A2 as described previously (53).

### Cotton rats.

Cotton rats (Sigmodon hispidus) were held at the animal facilities of Intravacc (Netherlands), and experiments were approved by the Animal Ethical Committee of RIVM. Animals were immunized intranasally with 10^5^ infectious units of rRSV or with uninfected Vero cell supernatant (mock) or intramuscularly with a high- or low-dose FI-RSV (1:5 and 1:125 diluted in phosphate-buffered saline [PBS], respectively) on days 0 and 21. Animals were challenged intranasally with 10^5^ infectious units of RSV-A2 on day 49. Animals were sacrificed, blood plasma was collected, and lungs were fixed on day 53. Histopathologic analysis of lung sections using hematoxylin and eosin staining was done by Paul Roholl (Microscope Consultancy, Netherlands) as described previously (54). To prepare single-cell suspensions, lung tissue of untreated animals was separated after DNase/collagenase digestion. Isolated lung cells were phenotyped by staining with antibodies to an epithelial marker, pancytokeratin (18), and T cell marker CD3ε (19).

### RSV infection and serology.

THP-1 cells, human PBMC, or cotton rat lung cells were used to measure ADE. Immune complexes were formed by preincubation of RSV with antibodies for 1 h at 37°C. Cells were inoculated by spinoculation for 1 h at 700 × *g* at 20°C in duplicate. Cells next were washed with PBS and replenished with culture medium. Overnight incubation at 37°C was followed by flow-cytometric analysis of GFP fluorescence using a FACSCanto II (BD Biosciences). RSV neutralization assays were carried out on Vero and Vero2a cells. Assays were performed in triplicate as described earlier (52). Fifty percent plaque reduction neutralization titers (PRNT50) were calculated from nonlinear regression using PRISM (GraphPad) after normalization to antibody-untreated, RSV-infected samples. Spinoculation had no effect on ADE or change of PRNT50s in any of the infection assays (data not shown). Antibody-binding sites of FCGRs were blocked by adding 3 μg/ml staphylococcal FLIPr-like protein (kind gift from Kok van Kessel, University Medical Center Utrecht) (55) to THP-1 cells during RSV neutralization assays. Cells were pretreated for 1 h and supplemented during infection and initial incubation steps. FLIPr-like protein was removed with the inoculum.

RSV-specific IgG concentrations in plasma samples were determined by enzyme-linked immunosorbent assay (ELISA) against RSV particles as described previously (6). IVIg at 1 mg/ml (KIOVIG; Baxter) was referred to as 1 arbitrary unit.
